# Mortality in Levodopa-Treated Parkinson's Disease

**DOI:** 10.1155/2014/426976

**Published:** 2014-01-28

**Authors:** John C. Morgan, Lillian J. Currie, Madaline B. Harrison, James P. Bennett, Joel M. Trugman, G. Frederick Wooten

**Affiliations:** ^1^Movement Disorders Program, Department of Neurology, Medical College of Georgia, Georgia Regents University, 1429 Harper Street HF-1154, Augusta, GA 30912, USA; ^2^Movement Disorders Division, Department of Neurology, University of Virginia, P.O. Box 800394, Charlottesville, VA 22908, USA; ^3^Parkinson's & Movement Disorders Center, Department of Neurology, Virginia Commonwealth University, 6605 West Broad Street, First Floor, Suite C Richmond, VA 23230, USA; ^4^Clinical Development, Forest Laboratories, Inc., Jersey City, NJ, USA

## Abstract

Parkinson's disease (PD) is associated with increased mortality despite many advances in treatment. Following the introduction of levodopa in the late 1960's, many studies reported improved or normalized mortality rates in PD. Despite the remarkable symptomatic benefits provided by levodopa, multiple recent studies have demonstrated that PD patients continue to die at a rate in excess of their peers. We undertook this retrospective study of 211 deceased PD patients to determine the factors associated with mortality in levodopa-treated PD. Our findings confirm that PD is associated with increased mortality in both men and women. Unlike the majority of other mortality studies, we found that women have a greater reduction in lifespan compared to men. We also found that patients with early onset PD (onset at the age of 50 or before) have reduced survival relative to PD patients with later ages of onset. A final important finding is that survival is equal in PD patients treated with levodopa early (within 2 years or less of PD onset) versus later.

## 1. Introduction

Before the introduction of levodopa, mortality in idiopathic PD was 2.9 times higher than that of the general population adjusted for age, gender, and race [1]. When levodopa was introduced in the late 1960's, PD patients realized remarkable symptomatic benefits and in multiple subsequent cross-sectional studies levodopa appeared to ameliorate or normalize mortality rates in PD [2–7]. Some authors found that levodopa reduced PD mortality during the first 6 years of therapy, with mortality returning to near prelevodopa levels after 12 years of treatment [8]. As one author reports, however, the majority of studies over the past 30 years have demonstrated that levodopa treatment in PD does not normalize mortality rates and these patients continue to die at a rate in excess of their peers [9]. More recent reports (1999–2004) have demonstrated a standardized mortality ratio (SMR) in PD patients ranging from 1.52 in a community-based Norwegian study [10] to a relative mortality risk of 3.38 in door-to-door survey in Ilan County, Taiwan [11].

Some factors such as severity of Parkinson's symptoms [12] and the presence of dementia [12–14] appear to increase mortality risk in PD. Many other factors such as gender, age of onset, disease duration, or delay in initiation of levodopa therapy may also affect mortality. Defining factors associated with increased mortality in PD is important as these factors may help clinicians anticipate outcomes and may influence therapeutic decisions.

## 2. Patients and Methods 

The University of Virginia Movement Disorders Database was screened for deceased patients who were diagnosed with idiopathic PD and chronically treated with levodopa. Patients who were diagnosed with vascular or drug-induced parkinsonism, as well as Parkinson's plus syndromes, were excluded from this analysis. Two hundred and eleven deceased patients who were diagnosed clinically with PD were retrospectively identified from the database. Patients had died between the years 1984 and 2002 with the mean year of death being 1996. Patients were diagnosed with PD using UK Parkinson's Disease Society Brain Bank criteria [15]. Our historical clinical diagnostic accuracy is 91% based upon autopsy of our patient population (G. F. Wooten, unpublished data) and is similar to that reported by other groups [16]. Only patients treated with levodopa throughout the course of their illness were included in this study (*n* = 211). Each patient was evaluated at least once at our institution by a movement disorders-trained neurologist (JPB, MBH, JMT, or GFW). The majority of patients were followed throughout their illness until death. We determined the cause of death (COD) in 197 patients by the review of medical records, discussion with the treating physician(s), interview of the decedent's relatives, and review of autopsy findings (available in 16 patients—6 patients underwent neuropathological investigation only and 10 patients had complete autopsy). All autopsied patients met neuropathological criteria for PD. Cause of death was considered to be PD if the patient died of complications related to PD (e.g., falls or aspiration pneumonia).

For each patient in this sample, age at PD onset and year of PD onset were used to create an age-, race-, and gender-matched control from the U.S. population based upon annual U.S. Life Expectancy Tables (U.S. Center for National Health Statistics, *Vital Statistics of the United States*). For patients with PD onset or death after age 85, decennial U.S. Life Expectancy Tables were used. For example, if a Caucasian man had PD onset at age 62 in 1990, then the 1990 U.S. Life Expectancy tables were used to determine the expected lifespan of a Caucasian man who was at the age of 62 in 1990 to create a control for this patient. The expected lifespan of controls was compared to the actual lifespan of the patient sample for analysis. The probability of each patient dying given their age of onset and disease duration was also determined using the appropriate United States Life Tables as described by Diamond and Markham [4]. The number of expected deaths was determined by summing the individual probabilities of death for each patient as described by Diamond and Markham [4]. Mortality ratios were calculated as the ratio of observed to expected deaths.

The medical records of the deceased PD patients were also screened for levodopa treatment parameters: time from PD onset until initiation of levodopa therapy (L-dopa delay), duration of levodopa therapy (L-dopa duration), and maximum levodopa dose (L-dopa max). These parameters were available for 197 patients for L-dopa delay and L-dopa duration and 169 patients for L-dopa max.

Data were analyzed using chi-square analysis for categorical variables and Student's *t*-test or the Mann-Whitney *U* test for continuous variables. Kaplan-Meier analysis was used to estimate survival when comparing groups of patients. Logistic regression was performed to identify clinical characteristics that were associated with patients dying of PD or other causes. All deceased PD patients were stratified by cause of death into two groups (PD and not PD) and then, through logistic regression, the variables of age of onset, age at death, gender, disease duration, L-dopa delay, and L-dopa duration were examined. Statistical analysis was performed using SPSS for Windows (SPSS, Inc., Chicago, IL).

## 3. Results 

Table 1 displays the disease characteristics of the 211 deceased, levodopa-treated PD patients in the study. Two hundred and seven patients were Caucasian and four were African-American (data not shown in Table 1). Approximately two-thirds of the patients were men and there were no differences between men and women in the sample with regards to age of onset, age at death, or disease duration.

**Figure 1:**
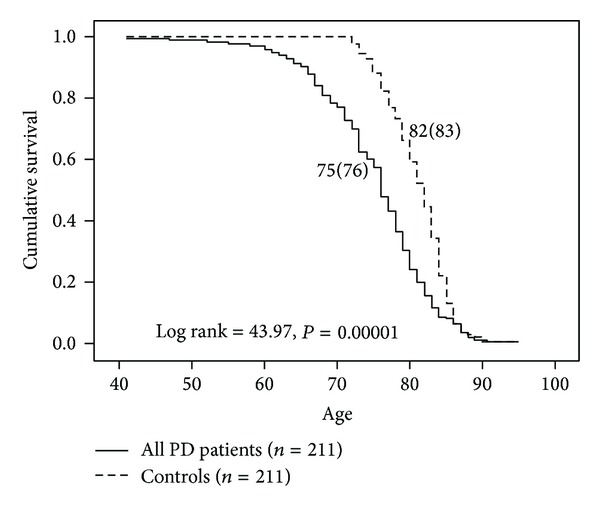
Survival analysis of all PD patients versus age-, gender-, and race-matched controls from the U.S. population. Controls had a seven-year longer survival on average relative to PD patients.

**Figure 2:**
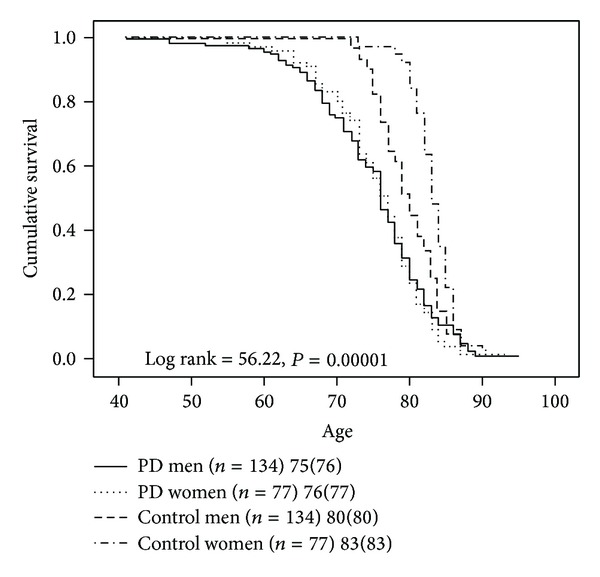
Survival analysis of PD men and PD women versus control men and control women from the U.S. population. PD men and PD women have no difference in survival, while both PD men and PD women have reduced survival relative to control men and control women, respectively.

A Kaplan-Meier survival analysis illustrated in Figure 1 reveals that survival is reduced on average by 7 years in the entire sample of PD patients compared to an age-, gender-, and race-matched control group from the U.S. population. Men with PD had a mean reduction in lifespan of 5 years relative to male controls while women with PD had their lifespan reduced by 7 years compared to female controls (Figure 2). Women with PD (*n* = 77) had a greater reduction in lifespan relative to men with PD (*n* = 134) (*P* < 0.001). There was no difference in survival between men and women with PD in our sample (Figure 2). When the probabilities of each patient dying were calculated as described by Diamond and Markham [4] using annual or decennial U.S. Life Tables, the overall observed to expected mortality ratio for the 211 PD patients was 2.66. Mortality ratios were 2.28 for PD men and 3.76 for PD women relative to age-, gender-, and race-matched controls from the U.S. population.

**Figure 3:**
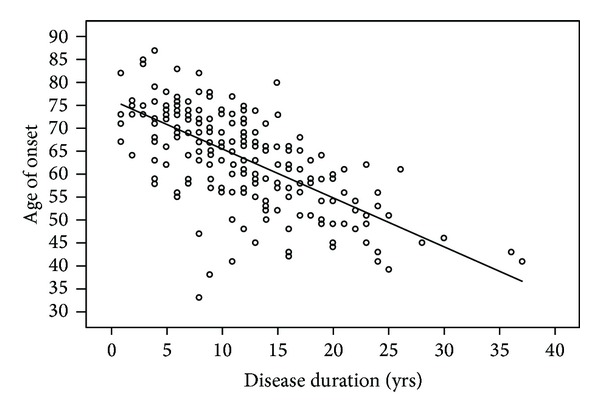
Scatter plot comparing age of onset to disease duration for each PD patient in our sample. Earlier age of PD onset was associated with longer disease duration in our sample as indicated by the trendline.

**Figure 4:**
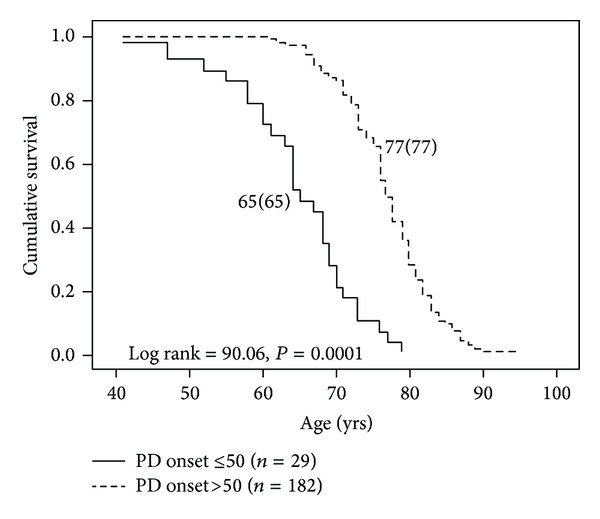
Survival analysis of PD patients with early age of onset (at age 50 or earlier) and later ages of onset. PD patients with early age of onset had markedly reduced survival (12 years) relative to the patients with later ages of onset.

Early age of onset (defined as PD onset at age 50 or earlier) was associated with longer disease duration in our sample (Figure 3). Patients with early age of onset (*n* = 29) were 12 years younger at death (65 ± 8 versus 77 ± 6, *P* < 0.001) and had a longer disease duration (20 ± 8 versus 11 ± 6, *P* < 0.001) when compared to patients with onset after age 50 (*n* = 182). Kaplan-Meier survival analysis revealed markedly reduced survival in early onset patients compared to patients with ages of onset after the age of 50 (Figure 4). Mortality ratios were 3.58 and 2.57, respectively, for the 29 patients with early PD onset versus the 182 patients with PD onset after the age of 50.

Cause of death by clinical impression (clinical history and medical records) was determined for 197 of the 211 patients and the frequencies for each cause of death are displayed in Table 2. About half of the patients died of PD-related causes with cardiovascular disease, cancer, and stroke causing the deaths in the remainder of the sample. There was no gender difference in the number of patients dying of PD-related causes versus non-PD causes (data not shown) (*P* = 0.315). When patients were categorized into intervals of 5 years disease duration, equal proportions of patients died of PD versus other causes irrespective of PD disease duration (data not shown) (*P* = 0.241).

Full autopsies were performed in ten patients and neuropathological diagnoses were available for six additional patients (Table 3). All patients met neuropathological criteria for PD at autopsy (16 of 16 patients) with two patients also meeting criteria for the diagnosis of diffuse Lewy body disease (DLBD) [17]. Two of these 16 patients also met CERAD criteria [18] for Alzheimer's disease in addition to their PD neuropathological diagnosis. The majority of fully autopsied patients died of bronchopneumonia (five of ten patients) with one patient dying of complications related to a hip fracture. This indicates that six of ten patients that underwent full autopsy died of PD-related causes.

Table 4 illustrates mortality in patients when stratified by age at death and disease duration. Four of eight patients dying at the age of 60 or earlier died of PD. The vast majority (83%) of patients died between the ages of 66 and 85. Eighty-nine percent of patients died within 20 years of their PD onset with only 11% of patients surviving with PD for more than 20 years. Equal proportions of patients died of PD regardless of age at death (*P* = 0.614) or disease duration (*P* = 0.244).

Table 5 displays levodopa treatment parameters for patients in the study. The delay from the time of PD onset until starting levodopa (L-dopa delay) and levodopa treatment duration (L-dopa duration) were available in 197 patients in the study. The levodopa maximum dose was available in 169 study patients. Levodopa maximum dose (L-dopa max) for patients in the study was equal in men and women. Kaplan-Meier survival analysis revealed equal survival in patients who started levodopa within the first two years of PD onset (*n* = 89) compared to those starting levodopa later (*n* = 108) (75(76) versus 76(76), *P* = 0.27).

## 4. Discussion 

This is one of the larger studies (211 patients) of mortality in PD since the levodopa era in PD began [1]. The major findings are as follows: (1) levodopa-treated PD patients appear to have excess mortality relative to controls from the U.S. population, (2) there does not appear to be a difference between men and women with PD with regard to death rate, but perhaps because control women live longer than control men, women with PD have increased mortality and a greater reduction in lifespan compared to men with PD, (3) patients with early PD onset (age 50 or earlier) appear to have increased mortality relative to patients with later PD onset, and (4) survival appears equal in PD patients treated with levodopa early (within 2 years of PD onset) versus later.

Methods for determining the effect of PD on mortality vary from retrospective to prospective, cross-sectional to longitudinal and may involve small cohorts or even entire populations. Despite differences in methodology, the vast majority of studies demonstrate that patients with PD continue to die at a rate in excess of controls [9]. Our results agree with these findings and demonstrate increased mortality in levodopa-treated PD patients relative to age-, race-, and gender-matched controls from the U.S. population. The most straightforward interpretation of these results is that levodopa, while effectively treating the motor symptoms of PD and improving life expectancy, does not normalize life expectancy in PD patients. Even though levodopa may improve motor symptoms in PD (tremor, bradykinesia, and rigidity), it may not slow disease progression [19]. As PD progresses, levodopa-resistant motor symptoms (speech/swallowing impairment, gait, and balance problems) and nonmotor symptoms (autonomic dysfunction, mood disorders, cognitive impairment, sleep disorders, and psychosis) become more prevalent and may contribute to increased morbidity and mortality [20]. Given the average disease duration of 12 years in our patient sample, it is likely that many of our patients suffered both levodopa-resistant motor and nonmotor symptoms that most likely contributed to increased morbidity and mortality.

The strengths of our study are (1) a well-defined and characterized sample of idiopathic PD patients with minimal contamination by other forms of parkinsonism, unlike some studies [21–23] and (2) a very large number of PD patients ascertained who were followed until death. A major limitation of this study is that the retrospective design may introduce a selection bias toward older patients or those with more prolonged or severe PD who were more likely to die. This weakness is partially mitigated by the very long ascertainment period of almost 20 years. In addition, the clinical characteristics of PD patients in our sample are similar to multiple retrospective/prospective studies with respect to age of PD onset, disease duration [2, 3, 7], and age at death [2, 24–26].

Another potential source of bias in our study is the setting of a specialty clinic in an academic medical center that might select for a population of patients with more severe or difficult to manage PD compared to community-based populations. It is important to note, however, that our retrospective deceased PD patient sample had a similar age of onset, disease duration, and age at death as studies described above in multiple clinical settings ranging from a single academic medical center [27] to a multicenter study [26], to a population-based study [28].

We observed a higher overall mortality ratio (2.66) in our retrospective deceased PD patient sample relative to some recently published prospective studies [29, 30]. The higher mortality ratio observed in our study may reflect the retrospective design bias given that at least two recent prospective studies have found much lower standardized mortality ratios of 1.1 in a Chinese cohort and 1.29 in a UK cohort [29, 30]. On the other hand, some prospective cohort- and population-based studies have recently found standardized mortality ratios ranging from 1.86 at 15 years to 3.1 at 20 years in the Sydney Multicentre Study of PD to as high as 3.6 in a population-based study of PD mortality in Bulgaria [31–33].

Our sample of deceased levodopa-treated PD patients shows a preponderance of men (64% of patients in the study). Similar to our results, most PD mortality studies have demonstrated an increased incidence in men [34–43]. This parallels our finding that men have 1.5 times the relative risk of developing PD based upon a meta-analysis of seven population-based incidence studies [44]. The reasons for the higher incidence of PD in men are unknown and might represent differences in genetic susceptibility [45], environmental exposure [46], head trauma, or increased female resistance to developing PD (perhaps due to the neuroprotective effects of estrogen) [47, 48].

Our data also demonstrate that women have higher mortality ratios compared to men (3.76 and 2.28, resp.) while having a similar age at death. This confirms the findings of Wooten et al. [44] and reflects a greater reduction in survival for women, given that women without PD typically live longer than men. This implies that women who get PD may lose (or have lost) putative longevity factors present in other women. This also indicates that PD progression is no different between women and men with respect to mortality.

Contrary to our findings, many authors found that men had greater mortality risk [34–43]. Other authors, however, found greater mortality risk in women in both pre-levodopa era [1] and more recently [7, 8, 49, 50]. Methodological differences in some of these studies may account for differences in mortality since sex-specific mortality tables were not used by some authors [39].

Complications of PD resulted in the death of over half of our patients (Table 2) similar to other studies [26, 34]. Autopsy results reveal almost 100% clinical diagnostic accuracy for PD in our group similar to other groups [16]. When patients were stratified by age at death and disease duration (Table 3), it is clear that PD contributed to the excess mortality of patients dying after the age of 50. This analysis also demonstrates that PD may contribute to many deaths regardless of disease duration. Patients with shorter disease durations dying of PD were older with presumably more comorbidities or may have had more aggressive disease [51].

Patients with early PD onset (age 50 or less) in our sample, while having a slower disease progression [51], actually died at a much younger age and had increased mortality relative to patients with ages of onset greater than 50. One prospective, cross-sectional study found similar mortality ratios in three groups of PD patients with mean ages of onset of 43, 55, and 66 years [51]. All patients in the three groups were followed for 17 years; however, patients with early age of onset in our study had a mean disease duration of 20 years, meaning that many of the early onset patients in the Diamond et al. [51] study probably had not died yet. Our finding of a higher mortality ratio in early onset PD patients is consistent with our ability to capture a greater number of early onset PD patients at death than if we followed them for 17 years. Because PD contributes to the death of similar ratios of early onset and later onset patients in our study, it is likely that PD significantly contributes to the increased mortality in an otherwise “healthier” age group when compared to older onset PD patients.

Supporting this, hypotheses are data from a more recent study of young-onset PD (YOPD) patients (defined as onset from age 21 to 40) [52]. The median age of deceased YOPD patients in the cohort was 57.7 years with a disease duration of 22.5 years while the median age of living YOPD patients was 50.1 years with a disease duration of 16.9 years [52].

Some in vitro studies have suggested that levodopa is toxic to dopaminergic neurons [53]. This has led to the hypothesis that levodopa might hasten progression of dopaminergic neuron death as well as morbidity and mortality in patients with PD. Our data indicate that survival was no better or worse in patients starting levodopa within two years of symptom onset versus greater than two years after symptom onset. The effect of levodopa on PD progression and mortality remains controversial despite its obvious symptomatic benefits for PD patients [54, 55].

The average disease duration for patients with PD in Hoehn and Yahr's seminal study published in 1967 was 9.4 years [1]. Thirty-five years after Cotzias et al. demonstrated that levodopa had long-term beneficial effects in PD [56], the average life expectancy for levodopa-treated PD patients after symptom onset is 12 years (in this study). The increase in survival could be due to the effects of levodopa therapy or other pharmacotherapies in PD, improved treatments for comorbid medical conditions such as cancer, diabetes, and heart disease, better and more available healthcare for patients, improved surgical treatments, and possibly healthier lifestyles.

## Figures and Tables

**Table 1:** Disease characteristics of deceased PD patients.

	*n*	Total sample (*n* = 211)		Men (*n* = 134) (64%)	Women (*n* = 77) (36%)	*P *
		Range	Mean (SD)	Mean (SD)	Mean (SD)	
Age of onset	211	33–87	64 (11)	64 (11)	63 (10)	0.60
Age at death	211	41–95	75 (8)	75 (8)	76 (7)	0.84
Disease duration	211	1–37	12 (7)	12 (7)	12 (7)	0.41

**Table 2:** Cause of death by clinical impression.

Cause of death	*n*	%
Parkinson's disease	111	52.6
Cardiovascular disease	26	12.3
Cancer	18	8.5
Stroke	14	6.6
Renal disease	9	4.3
Suicide	5	2.4
Respiratory disease/illness	4	1.9
Other neurological	4	1.9
Other medical	3	1.4
Diabetes	2	0.9
Old age	1	0.5
Unknown	14	6.6

**Table 3:** Neuropathology and cause of death by autopsy.

Neuropathology (*n* = 16)	*n*
Parkinson's disease	12
Diffuse Lewy body disease	2
Parkinson's disease and Alzheimer's disease	2

Cause of death (*n* = 10)	*n*

Bronchopneumonia	5
Breast cancer	1
Lymphoma	1
Myocardial infarction	1
Hip fracture	1
Unknown	1

**Table 4:** Mortality in PD patients stratified by age at death and disease duration.

Age at death	Percentage of total dead	Observed deaths	Expected deaths	Mortality ratio	Number of PD deaths
41–45	0.5	1	0.0189	52.91	0
46–50	0.5	1	0.0263	38.02	0
51–55	0.9	2	0.0778	25.71	1
56–60	1.9	4	0.6277	6.37	3
61–65	5.7	12	1.5998	7.50	5
66–70	13.3	28	6.3265	4.43	11
71–75	19.9	42	11.9488	3.52	24
76–80	33.2	70	26.5476	2.64	39
81–85	16.1	34	18.5483	1.83	20
86–90	7.6	16	12.5786	1.27	7
91–95	0.5	1	1.0000	1.00	1

Disease duration	Percentage of total dead	Observed deaths	Expected deaths	Mortality ratio	Number of PD deaths

1–5	17.5	37	7.0770	5.23	16
6–10	29.4	62	20.7956	2.98	28
11–15	25.6	54	23.7313	2.28	34
16–20	16.6	35	15.9910	2.19	22
21–25	8.1	17	7.7683	2.19	8
26+	2.8	6	3.9371	1.52	3

**Table 5:** Levodopa treatment parameters.

	*n*	Total sample (*n* = 211)		Men		Women		*P*
		Range	Mean (SD)	*n*	Mean (SD)	*n*	Mean (SD)	
L-dopa delay	197	0–15	2 (3)	124	2 (3)	73	3 (3)	0.31
L-dopa duration	197	1–30	9 (6)	124	9 (6)	73	9 (6)	0.66
L-dopa max	169	150–2250	833 (429)	108	870 (441)	61	767 (403)	0.16

L-dopa delay: years from PD onset until L-dopa initiation; L-dopa duration: years of L-dopa treatment; L-dopa max: maximum recorded L-dopa dose in milligrams.
